# Registered reports in forensic science

**DOI:** 10.1098/rsos.221076

**Published:** 2022-11-30

**Authors:** M. M. Houck, J. Chin, H. Swofford, C. Gibb

**Affiliations:** ^1^ Graduate Program Director, Global Forensic and Justice Center, Florida International University, Miami, FL 33199, USA; ^2^ College of Law, Australian National University Sydney, Sydney, NSW 2000, Australia; ^3^ HJS Consulting, LLC, Washington, DC, USA; Senior Editor, Forensic Science International: Synergy; ^4^ The University of Twente, Amsterdam, The Netherlands

**Keywords:** registered reports, forensic science, quality, methodology, justice

## Abstract

Research assessing the validity and reliability of many forensic science disciplines has been published; however, the quality of this research varies depending on the methodologies employed. This was a major point of contention with the United States' President's Council of Advisors on Science and Technology, who recognized the existing literature but found the majority lacking because of methodological issues. Questionable scientific methodologies have undermined the forensic science community's ability to defend the scientific foundations and examination protocols used to examine evidence in criminal cases. Such scientific failures have significant legal implications. Registered reports, which strengthen the quality of scientific research and reliability of laboratory protocols, can provide transparency, validity and a stronger scientific foundation for forensic science.

## 1. Forensic methods in question

Forensic science is a cornerstone of advancing investigations or establishing facts in question to support criminal or civil litigation. Interpretations and conclusions made by specialists following forensic examinations have life-changing consequences for individuals and society. Thus, it is imperative that interpretations and conclusions are based on sound scientific principles, methods and practices. Over the years, increasing concerns have been raised within the scientific community as it relates to the validity and reliability of scientific methodologies underpinning various forensic science disciplines [1,2]; for example, ‘PCAST found few black-box studies appropriately designed to assess scientific validity of subjective methods’ [1, p. 68]. Although thousands of research studies related to forensic science methods and applications have been published in peer review journals over the years [3], the validity of these studies varies depending on the rigour of the underlying scientific methodology employed.

Issues concerning the quality of existing research supporting forensic science became a key point of contention in 2009, when the National Research Council (NRC) raised the question of the extent to which there is *science* in any given forensic science discipline [2] and again in 2016, the President's Council of Advisors on Science and Technology (PCAST) [1] applied a critical lens to the existing body of research often referenced by the forensic science community as the bedrock of their examination methodologies and was largely dismissive of a significant proportion of studies intended to assess the accuracy and reliability of many forensic disciplines [1]. Many within the forensic science community were quick to publicly disagree with the findings from PCAST and reaffirm the credibility of forensic science methods, including several major forensic science professional organizations (e.g. [4–6]). They took issue with the PCAST's decision to discount and ignore research on methodological grounds, such as small sample sizes, and lack of open-set, black-box designs. Although significant negative reactions across the forensic science community to the PCAST report caused the PCAST to reflect on their findings and recommendations and re-engage relevant stakeholders, they ultimately stood firm in their assessments [7]. These concerns are not limited to just the PCAST. More recently, controversy has ensued as it relates to the methodological design and calculation of error rates, specifically how ‘inconclusive’ decisions are accounted for in error rate studies [8–15]. These concerns have raised questions as to whether courts and the public have been misled when presented with statistics touting the reliability of forensic evidence presented in courts.

Irrespective of whether one agrees with the PCAST and others within the scientific community that have been critical of the methodological design of forensic science research, the controversy has nevertheless cast a shadow over the credibility of many forensic science disciplines. This has caused some litigators and courts to question the reliability of many traditional forensic science practices and challenge the admissibility of forensic evidence. This is a problem that cannot be addressed through mere arguments and critiques in response. Instead, it will require a fundamental shift in how forensic science research is approached.

## 2. Reliable methodologies underpin legal admissibility

One of the most important aspects of admission and reliance on forensic evidence in criminal trials is the extent to which a forensic discipline is founded on a reliable scientific methodology [2]. However, in 2009 the NRC noted that while ‘[f]orensic science examiners need to understand the principles, practices and contexts of scientific methodology, as well as the distinctive features of their specialty’, they ‘face pressure to sacrifice appropriate methodology for the sake of expediency’ [2, pp. 23, 24, 27]. This environment flies in the face of statutes and rulings that emphasize the court's focus only on the expert's foundational principles and applied methodology and not on any conclusions that result from them. Expert conclusions and opinions based on a methodology that diverges significantly from the procedures accepted by recognized authorities in the field cannot be generally accepted as a reliable technique [2].

For example, US Federal Rule of Criminal Procedure, 16 A(1)G states,

At the defendant's request, the government must provide a written summary of any testimony that the government intends to use under Rules 702, 703, or 705 of the Federal Rules of Evidence during its case-in-chief at trial. If the government requests discovery under subdivisions (b)(1)(C)(ii) and the defendant complies, the government must, at the defendant's request, give to the defendant a written summary of testimony that the government intends to use under Rules 702, 703, or 705 of the Federal Rules of Evidence as evidence at trial on the issue of the defendant's mental condition. **The summary provided under this subparagraph must describe the witness's opinions, the bases and reasons for those opinions, and the witness's qualifications** (emphasis added) [16].

Federal Rule of Evidence 702 states, in part,

A witness who is qualified as an expert by knowledge, skill, experience, training, or education may testify in the form of an opinion or otherwise if…**(c) the testimony is the product of reliable principles and methods; and (d) the expert has reliably applied the principles and methods to the facts of the case** (emphasis added) [17].

The USA is not unique in these requirements. The Supreme Court of Canada has adopted Rule 702's reliability requirements in its expert evidence doctrine (*White Burgess Langille Inman v Abbott and Haliburton Co*, 2015). Similarly, several Australian jurisdictions require expert witnesses to provide the court with the ‘necessary scientific criteria for testing the accuracy’ of their opinions as a requirement for admitting that evidence (*Makita Pty Ltd v Sprowles*, 2001).

As a result, forensic science is on the front line of society's current ‘crisis of expertise’ [18]. In other words, many fields struggle to maintain legitimacy in the face of politically motivated attacks on scientific findings. Legitimacy takes on a different meaning and level of importance in forensic science, in which sound, valid methodologies enhance the credibility of the field to courts and the public. As a public good, the public expects transparency and no reporting biases in science that inform the criminal justice process. Exaggerating findings repeatedly have consequences for their credibility, regardless of their veracity [19]. Once outside the laboratory, the conclusions are in the hands of lawyers and judges, who often have insufficient training and background in scientific methodology. Legal actors often fail to fully comprehend the approaches employed by different forensic science disciplines and the reliability of forensic science evidence offered in trials [2]. Therefore, it is up to the scientific community, and forensic science community specifically, to ensure that methodologies are sound and valid before being applied in casework. How best to vet the myriad of methods used by forensic service providers worldwide? We contend that the methodology, the bedrock of any science's legitimacy and reliability, presents a way forward via registered reports.

## 3. Registered reports

Registered reports are a publishing format that promotes rigorous methodology and adherence to that methodology by conducting peer reviews prior to the results being known. Authors submit ‘Stage 1’ manuscripts (including theory, hypotheses, methods and analysis plans) prior to collecting data—or looking at the data if the data are pre-existing. Stage 1 manuscripts are peer-reviewed based on methodological quality and their ability to address research questions. If they pass this process, then the ‘Stage 2’ manuscript (the approved protocol plus results) will be published if the authors follow through with the accepted methodology.

The registered report format is designed to reward best practices in the design of transparent, reproducible methods which lead to unambiguous reporting, regardless of the outcome. It eliminates a variety of questionable research practices by removing the incentive to selectively report results and increasing transparency such that selective reporting would be easy to detect. The format also allows reviewers to help improve the methods by suggesting more appropriate statistical models and encouraging higher powered designs, among other benefits.

Early research on registered reports suggests that these salutary aims have been achieved. Registered reports are associated with more realistic proportions of positive results [20] and a perceived higher quality of research [21]. As a result, they seem to improve research quality, while reducing publication bias. More than 300 journals now accept registered reports [22]. Recently, *PLoS Biology* observed an 88% acceptance rate for registered reports with substantial revisions for most submissions, compared to 50% acceptance in its regular track [23]. The 88% rate is possibly due to reviewers being able to improve the methods during peer review and ensure that the methodologies are sound from the outset prior to the expended resources and research being conducted. Registered reports seem to improve the quality and credibility of the published literature in several fields.

## 4. Registered reports as a tool for improving validated methods

While forensic practitioners focus on daily operational activities, many are motivated to involve themselves in scientific research and development as well as validation studies required for accreditation and admissibility. Depending on their organization and the scientific health and cultural changes within their organizations, practitioners may have limited access to qualified collaborators. At the same time, practitioners bring an important perspective with direct access to specialist tools, other qualified practitioners in allied disciplines and expertise. How these projects are designed, what is being performed, and the results, let alone their validity, remain largely unknown and less defensible than those with more transparent methodological designs.

**Figure 1.**
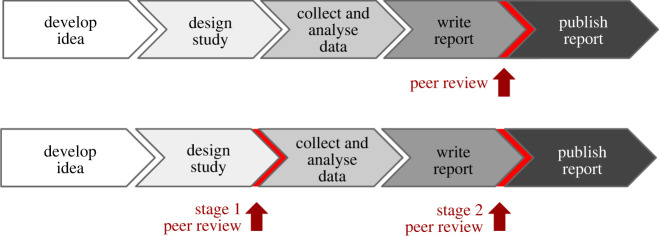
Traditional research publication process and the registered report protocol compared. Redrawn from: https://osf.io/rr.

Notably, technical guidelines regarding the performance of validation studies have been published for practitioners working in operational environments (e.g. ENFSI and UK Regulator)^1^; however, in terms of the quality, validity and reliability of the methods applied and the resulting published results, in-house studies still lack scientific accountability. Registered reports provide accountability and, more importantly, from the practitioner's perspective, they provide a set of clear instructions to ensure a robust and more defensible approach (for a complete overview of the process, see https://osf.io/rr/) (figure 1). Although, at first glance, it may seem like a daunting task, what the field should aim for is encouraging a more transparent, consistent platform that promotes scientific integrity. Currently, countless in-house unpublished validation studies and other research and development studies are hidden or lost behind organizational lines. This would serve the community to have a better and more transparent process to ensure that the results of these studies are published for a wider forensic community. The registered report manuscript would be published regardless of the study's outcomes; this feedback is crucial for forensic science to move forward. Publication of the results requires a valid study design. Registered reports provide this information.

## 5. Registered reports can improve forensic methods and justice outcomes

*Forensic Science International: Synergy* was the first journal in forensic science to adopt registered reports^2^. Part of the journal's mission is that because forensic services are sciences integral to a just society governed by the rule of law, they are unarguably a public good and should be accessible to anyone. Open, transparent science helps to promote justice, and we believe that registered reports support this mission.

To this end, registered reports can provide more reliable results through the vetting of methods prior to data collection, decreasing the realistic proportion of positive results and addressing issues with method quality before conclusions are drawn. Moreover, the registered reports are in line with many laws that provide methodological transparency in the justice process. For example, the ability to fully examine evidence tendered against an accused is a component of the right to fair trial in many jurisdictions. For example, in the United States, the Sixth Amendment of the Constitution provides that a person accused of a crime has the right to confront a witness against them in a criminal action. This ensures that witnesses testify under oath and understand the serious nature of the trial process, allows the accused to cross-examine witnesses who testify against them, and, crucially for registered reports, allows jurors to assess the credibility of a witness. The witness's credibility, in large part, stems from the validity of their methods and the conclusions drawn from them. On the stand, the witness would be able to discuss the vetting of the method, its increased validity through the registered reports process, and thus be better seen as the neutral arbiter of science, rather than an advocate using untested methods only they can make work; see [24] for a particularly egregious example of a lack of validation.

Several jurisdictions also exclude evidence when its probative value is exceeded by the danger of unfair prejudice [25]. The probative value is undermined by poor methodology and a lack of methodological transparency. Methodological opacity also makes evidence untestable by the adverse party, heightening its unfair prejudice and potential exclusion if it infringes on due process (e.g. relating to the Fifth and Fourteenth Amendments to the Constitution).

## 6. Conclusion

In summary, registered reports can provide a strong evidence base for forensic science. They align with principles of justice, such as the right to examine and confront evidence and the purposes that underlie the rules of evidence in many jurisdictions. Researchers, editors and other stakeholders can sign an open letter^3^ to help bring registered reports to other forensic science journals. The profession and discipline of forensic science must demonstrate validity and transparency to its stakeholders and the public it serves.

## Data Availability

No data supporting this work were generated.

## Footnotes

1 See https://enfsi.eu/wp-content/uploads/2017/06/Guidance-QCC-VAL-002.pdf and https://assets.publishing.service.gov.uk/government/uploads/system/uploads/attachment_data/file/920449/201_-_FSR-G-201_Validation_Guidance_Issue_2.pdf as examples.

2 https://www.elsevier.com/__data/promis_misc/FSI%20Synergy%20-%20RR%20author%20guidelines.pdf.

3 https://www.jasonmchin.com/jason-chin-evidence-blog/2020/7/31/help-us-bring-registered-reports-to-forensic-science.
